# Emerging therapeutic strategies for cystinosis

**DOI:** 10.3389/fped.2025.1601409

**Published:** 2025-05-21

**Authors:** Paul Goodyer, Elena Torban

**Affiliations:** ^1^ Department of Pediatrics, McGill University, Montréal, QC, Canada; ^2^ Research Institute of the McGill University Health Centre, Montréal, QC, Canada; ^3^ Department of Medicine, McGill University, Montréal, QC, Canada

**Keywords:** cystinosis, therapeutic strategies, mRNA, pro-drug, gene therapy, flavinoids

## Abstract

For over 40 years, oral cysteamine has been the mainstay of therapy for cystinosis. While it has been of great benefit, slowing organ deterioration and prolonging life, cysteamine is not well tolerated and may not rescue all pathogenic mechanisms driving the disease. Of late, research groups around the world have been pursuing various novel therapeutic strategies. Here we select just four of many examples - two that address events downstream of the missing Cystinosin protein and two that aim to address the upstream CTNS mutation. Our aim is to update the cystinosis community on some of the exciting work in progress. We have drawn from preliminary reports and oral presentations at cystinosis meetings. While each approach requires further work and critical analysis, the sheer number and variety of these potential therapies is cause for hope.

## 1 Introduction

In 1977, Crawhall, Thoene and Schneider discovered that the pathologic accumulation of cystine in cells from cystinosis patients could be reversed by cysteamine treatment—thereby bypassing the mutant lysosomal cystine transporter, known as Cystinosin, encoded by the *CTNS* gene (1). They showed that the therapeutic mechanism involves chemical reduction of intralysosomal cystine to yield small mixed disulfides that exit the lysosome via an alternative amino acid transporter (PQLC2). In 1987, a multinational clinical trial in 93 cystinosis patients showed that oral cysteamine could reduce leukocyte cystine levels by about 85% and slowed progressive renal insufficiency, if started early in life (2). Subsequent studies confirmed that oral cysteamine slows deterioration of the kidneys and other organs by 5–10 years. This drug has served as the mainstay of cystinosis therapy for the last 40 years.

However, after 4 decades, further therapeutic breakthroughs have been long-overdue. Although the rigid 6-hourly dosing schedule for cysteamine was overcome by the development of a delayed-release form of the drug, most cystinosis patients still develop renal Fanconi Syndrome in the first years of life, still eventually require renal transplantation and still face a shortened life-span. The partial efficiency of cysteamine can be attributed to suboptimal adherence to oral cysteamine due to its unpleasant side-effects. However, careful analysis of the data from the retrospective study by Brodin-Sartorius et al. suggests that nearly a third of cystinosis patients develop early (<12 years) renal insufficiency despite the start of the cysteamine treatment before the age of 3 years (3). Studies from several groups now confirm that the cystinosin protein may have important cellular functions (e.g., autophagy) in addition to its role as a lysosomal cystine transporter; these functions may be beyond the reach of cysteamine (4).

Several groups have recognized the importance of the crystal-induced inflammatory cascade in cystinosis. Elmonem and colleagues summarized the underlying biology, involving release of interleukins and other proinflammatory signals from tissue macrophages as they engulf cystine crystals (5). There are now new selective anti-inflammatory drugs with good biosafety profiles that offer alternatives to NSAIDs for suppression of subpathways in the complex inflammatory cascade. However, Elmonem points out that the potent NSAID, indomethacin, has been used widely in children with cystinosis over many years and there has been no indication that this quells the progression of renal insufficiency. Mak and colleagues have reported an interesting effect of high-dose 25(OH) Vitamin D on cachexia and muscle wasting in *Ctns* mutant mice (6). However, the doses used were about ten times that associated with Vitamin D3 toxicity (15,000 IU/day) in humans. For this to be translated to clinical care, the underlying therapeutic mechanism must be understood and a safer alternative must be identified.

Fortunately, the roux has been stirred and the pot seems to be boiling once again around cystinosis. Clinical care is evolving toward a multidisciplinary approach, beyond the traditional domains of the nephrologist or the biochemical geneticist. Similarly, worldwide efforts now embrace a broad range of novel therapeutic strategies that reach beyond the time-honored standard of care. In this brief review, we summarize only a sample of the many emerging therapeutic strategies that offer new hope for cystinosis patients. These include the prospect of a better-tolerated cysteamine prodrug and specific dietary nutrients that target “downstream” pathways. Other potential therapies focus on “upstream” approaches which aim to replace the mutant CTNS gene in patient cells or deliver normal CTNS messenger RNA to tissues via lipid nanoparticles. While our goal is to update the cystinosis community on recent (in several cases unpublished) progress, it should be noted that all ideas require further testing and/or peer-reviewed clinical trials.

## 2 Downstream Approaches

### 2.1 Cysteamine prodrug

In 2008, the Cairns research group at the Robert Gordon University (Aberdeen, UK) screened a series of “prodrug” variants of cysteamine, with the aim of overcoming the offensive smell of degradation products (dimethylsulfide and methandiol) that make it difficult to sustain adherence to long-term oral cysteamine therapy (7). Inspired by this early progress, the challenge was taken up by Rosleen Anderson's team at the University of Sunderland who reported a novel prodrug with an esterified amino terminus that improved gastrointestinal absorption, a terminal thioester to block rapid generation of the offensive thiol compounds and a glutamate moiety preceding cysteamine to target metabolism of the prodrug at the surface of cells in tissues such as the kidney (8). With the untimely death of Dr. Anderson in 2018, the project was put at risk. However, her colleagues pushed forward to spawn a small biotech company (https://sundarapharma.com/cf10-cystinosis/) that continues to develop the lead compound, CF10. A key preclinical CF10 study in a rat model of cystinosis has been performed by Jennifer Hollywood et al. intended for peer-reviewed publication this year (personal communication). A Phase I dose-finding clinical trial will be underway in 2025 at the University Hospitals Birmingham (PI: Graham Lipkin), engaging cystinosis patient volunteers (personal communication). Data from the company website (not peer-reviewed) suggest that the prodrug is effective in lowering cell cystine *in vitro* and is well-absorbed and well-tolerated in rats and dogs. Preclinical pharmacokinetic studies showed relatively low serum levels of free cysteamine but excellent uptake of prodrug in tissues, compatible with twice-a-day dosing. Infusion of the prodrug into dogs generated no significant dimethylsulfide in blood.

*While careful analysis of efficacy, tolerability and safety remains to be confirmed in future clinical trials, the data thus far are promising and offer reason for cautious optimism.*

### 2.2 Novel therapeutic strategies to rescue defective autophagy in cystinosis

In 2010, Sarwal's team from the University of California San Francisco reported that renal proximal tubular cells from cystinosis patients exhibit an abnormal accumulation of autophagosomes identified by the biomarker, LC3 (9). Antignac's group from the Imagine Institute in Paris then showed that cystinosin protein directly interacts with the mTORC1 protein complex at the lysosomal wall that controls inward nutrient flux (10). Hollywood et al. studied this process in induced pluripotent stem cells from cystinosis patients and demonstrated that mutant cells accumulate large, abnormal autophagosomes that appear to be arrested in the final stages of cargo transfer between autophagosomes and lysosomes (4). They observed that inhibitors of the mTORC1 pathway (e.g., everolimus) could reverse the defect in autophagosome cycling and might be used in combination with cysteamine to treat cystinosis. However, the details of this pathway are not fully understood, and it is not yet clear whether mTORC1 inhibitors will be useful in treating cystinosis.

On the other hand, Emma's group in Bambino Gesù Children's Hospital, Rome, discovered that genistein, a naturally occurring flavinoid found in soy beans, was able to decrease cystine accumulation in CTNS mutant cells (11). They proposed that it did so by inducing a master transcription factor (TFEB) for lysosomal pathways, including autophagosome cycling and lysosomal exocytosis (11). When *Ctns(**−/−)* mice were fed a diet high in genistein, they observed decrease in kidney cystine and improved renal function (12).

*Although still in the preclinical stage, these observations offer the intriguing possibility that an approved drug used in transplantation or naturally-occurring flavinoids may be useful in treating the autophagy defect in cystinosis which cannot be addressed by cysteamine.*

## 3 Upstream approaches

### 3.1 Transplantation of genetically-modified hematopoietic stem and progenitor cells

In 2009, at a Cystinosis Research Network meeting in Atlanta GA, Stephanie Cherqui reported her surprising observation, that transplantation of hematopoietic stem and progenitor cells (HSPCs) from wildtype mice into homozygous *Ctns**−/−* mutant after chemical bone marrow ablation resulted in repopulation of the marrow followed by progressive accumulation of the wildtype cells in the mutant kidney interstitium and other organs (13). Pathologic kidney cystine levels were reduced to about 43% of that in the mutant organ. It was initially thought that the exogenous hematopoietic cells replaced or fused with mutant cells in cystinotic organs, but it was later shown that circulating wildtype HSPC-derived monocytes had taken up a perivascular position, adjacent to the intrinsic nephron cells, transferring mRNA, protein and whole lysosomes in paracrine fashion via tunneling nanotubes (14, 15).

In 2019, Cherqui launched a clinical trial applying this strategy in six young adult patients with nephropathic cystinosis after FDA approval and issuance of an IND (16).

Patient CD34+ (monocyte lineage) HSPCs were harvested and genetically modified by insertion of a normal CTNS cDNA (driven by a moderately potent promoter) into a “safe harbor” site within the genome, using a self-inactivating lentivirus. Following partial ablation of the patient's bone marrow with busulfan, the genetically-modified autologous cells were re-infused into each patient.

This clinical trial is still ongoing and long-term results are still awaited. However, several promising observations have been reported by Cherqui at various meetings of the Cystinosis community. Successful (but variable) long-term reconstitution of patient bone marrow by genetically-modified HSPCs was achieved in all six subjects. Four of the six patients have remained off cysteamine therapy. The two patients without prior kidney transplantation showed continued loss of function in the native kidney, but this is difficult to interpret because autologous HSPC transplantation was performed at a relatively late stage of kidney deterioration. On the strength of the findings from this clinical trial, Novartis Pharmaceuticals recently announced that they would conduct a new multi-centre clinical trial of genetically- modified autologous HSPCs in younger children with nephropathic cystinosis and good renal function.

*While it is premature to evaluate the success of this therapy in cystinosis, the upcoming trial in young children holds great interest.*

### 3.2 Repurposing of the COVID vaccine messenger RNA technology for therapy of cystinosis

In 2020, the onset of the COVID-19 pandemic cast a dark shadow on many aspects of biomedical research, but the metaphor of John Milton in 1634, “Every cloud has a silver lining” may be applicable in the case of cystinosis. It is possible that the lipid nanoparticle (LNP)/mRNA technology used to rapidly develop COVID-19 vaccines (expression of COVID spike protein mRNA in the host) could be re-purposed to deliver normal CTNS mRNA to tissues affected by cystinosis. The strategy would involve repeated injection of LNP/CTNS mRNA to bypass the loss of the CTNS gene for limited periods.

In 2023, Elena Levtchenko's group (Leuven, Belgium) transfected both mutant renal proximal tubular cells and podocytes from a cystinosis patient with a stabilized HA-tagged CTNS mRNA designed by RiboPro B.V. in the Netherlands. Cystine accumulation was reduced by 75% for 4 days before it began to slowly reaccumulate over the next 10 days in the tubular cells and after 18 days in podocytes (17). Furthermore, direct injection of the mCherry-tagged *CTNS* mRNA into *Ctns**−/−* zebrafish embryos reduced cystine accumulation in the animals and improved tubular retention of an injected low-molecular weight dextran marker, suggesting partial rescue of defective proximal tubular function.

Similarly, our group (McGill University in Montreal) transfected mutant renal proximal tubular cells *in vitro* with a stabilized V5-tagged *CTNS* mRNA generated by Moderna Pharmaceuticals. We noted normalization of both cell cystine levels and autophagosome accumulation. This mRNA has now been formulated by Moderna into a novel LNP for testing in *Ctns* mutant mice; these studies are ongoing and await peer review.

*The potential application of LNP/mRNA therapy to cystinosis is still at an early stage and it remains to be seen whether modified LNPs can deliver sufficient mRNA to cystinosis-relevant tissues to rescue all primary features of the disease (defective cystine efflux from the lysosome and defective autophagosome trafficking to the lysosome). However, lessons from COVID-19 vaccine technology may offer an unanticipated “upstream” strategy for treatment of cystinosis.*

## 4 Concluding remarks

This review has focused on four of research areas in which investigators are exploring novel approaches to therapy of cystinosis. Of these, the autotransplantation of genetically-modified HSPCs is furthest advanced. However, it is clear that the investment in therapeutic research by the cystinosis community has fermented a wide range of possibilities that offer great hope for the future.
